# Efficient CRISPR/Cas9 Genome Editing of *Phytoene desaturase* in Cassava

**DOI:** 10.3389/fpls.2017.01780

**Published:** 2017-10-18

**Authors:** John Odipio, Titus Alicai, Ivan Ingelbrecht, Dmitri A. Nusinow, Rebecca Bart, Nigel J. Taylor

**Affiliations:** ^1^Donald Danforth Plant Science Center, St. Louis, MO, United States; ^2^National Crops Resources Research Institute, Kampala, Uganda; ^3^Vlaams Instituut voor Biotechnologie, Department of Plant Biotechnology and Bioinformatics, Faculty of Sciences, Ghent University, Ghent, Belgium; ^4^FAO/IAEA Division of Nuclear Techniques in Food and Agriculture, Department of Nuclear Sciences and Applications, International Atomic Energy Agency, Vienna, Austria

**Keywords:** cassava, genome editing, CRISPR/Cas9, *Phytoene desaturase* (*PDS*), albino, mutation, homozygous, heterozygous

## Abstract

CRISPR/Cas9 has become a powerful genome-editing tool for introducing genetic changes into crop species. In order to develop capacity for CRISPR/Cas9 technology in the tropical staple cassava (*Manihot esculenta*), the *Phytoene desaturase* (*MePDS*) gene was targeted in two cultivars using constructs carrying gRNAs targeting two sequences within *MePDS* exon 13. After *Agrobacterium*-mediated delivery of CRISPR/Cas9 reagents into cassava cells, both constructs induced visible albino phenotypes within cotyledon-stage somatic embryos regenerating on selection medium and the plants regenerated therefrom. A total of 58 (cv. 60444) and 25 (cv. TME 204) plant lines were recovered, of which 38 plant lines (19 from each cultivar) were analyzed for mutagenesis. The frequency of plant lines showing albino phenotype was high, ranging from 90 to 100% in cv. TME 204. Observed albino phenotypes were comprised of full albinos devoid of green tissue and chimeras containing a mixture of white and green tissues. Sequence analysis revealed that 38/38 (100%) of the plant lines examined carried mutations at the targeted *MePDS* site, with insertions, deletions, and substitutions recorded. One putatively mono-allelic homozygous line (1/19) was found from cv. 60444, while 1 (1/19) and 4 (4/19) putatively bi-allelic homozygous lines were found in 60444 and TME204, respectively. The remaining plant lines, comprised mostly of the chimeras, were found to be putatively heterozygous. We observed minor (1 bp) nucleotide substitutions and or deletions upstream of the 5′ and or downstream of the 3′ targeted *MePDS* region. The data reported demonstrates that CRISPR/Cas9-mediated genome editing of cassava is highly efficient and relatively simple, generating multi-allelic mutations in both cultivars studied. Modification of *MePDS* described here generates visually detectable mutated events in a relatively short time frame of 6–8 weeks, and does not require sequencing to confirm editing at the target. It therefore provides a valuable platform to facilitate rapid assessment and optimization of CRISPR/Cas9 and other genome-editing technologies in cassava.

## Introduction

Development of superior crop varieties has relied on securing genetic gains through sexual recombination, induced random mutagenesis, and transgenic approaches. The recent emergence of targeted genome-editing technologies offers a new avenue for incorporating beneficial genetic changes in the world’s most important crop species (Zhang et al., 2017).

Gene-editing technologies based on zinc-finger nucleases (ZFNs), transcription activator-like effector nucleases (TALENs), and clustered regularly interspaced short palindromic repeats (CRISPR) make use of engineered nucleases to induce double strand breaks (DSB) at known DNA sequences within the genome. Subsequent repair at the target site introduces variation via error prone non-homologous end joining (NHEJ) (Sonoda et al., 2006; Curtin et al., 2012; Joung and Sander, 2013) to generate insertions and deletions (INDELs), with occasional substitution of nucleotides occurring at the repair regions. Alternatively, the error free homologous recombination (HR) repair pathway can be activated in presence of a homologous donor DNA template spanning the DSB, resulting in targeted gene replacement (Puchta et al., 1996; Johnson and Jasin, 2001; Symington and Gautier, 2011).

The ZFNs and TALENs are based on protein-guided recognition mechanisms. Vector construction for targeting DNA sequences using these technologies is a relatively complex and costly process. In contrast, the CRISPR/Cas9 system depends on DNA or RNA sequence homology, in which targeting a specific DNA sequence requires only a 17–23-bp complementary nucleotide sequence called a guide RNA (gRNA). This versatility, plus high efficacy, and low cost have made CRISPR/Cas9 the most widely adopted of the three genome-editing technologies (Doudna and Charpentier, 2014). CRISPR/Cas9 has been applied to delete, activate, and suppress targeted genes in human cells, animals, and plants (Pennisi, 2013). Plant species altered by CRISPR/Cas9 technology include *Nicotiana benthamiana* (Nekrasov et al., 2013), Arabidopsis, tobacco, sorghum, and rice (Jiang et al., 2013), tomatoes (Brooks et al., 2014), wheat (Shan et al., 2013), soybean (Sun et al., 2015), maize (Svitashev et al., 2016), potato (Wang et al., 2015), sweet orange (Jia and Wang, 2014), populus (Fan et al., 2015), cucumber (Chandrasekaran et al., 2016), and cotton (Li et al., 2017). In several cases, CRISPR/Cas9-induced mutations have been confirmed to be heritable across sexual generations (Brooks et al., 2014; Ding et al., 2016).

Cassava (*Manihot esculenta* Crantz) is an essential source of calories for millions of people across the world’s tropical regions, providing storage roots for local consumption and raw materials for processed foods and industrial products (Ceballos et al., 2015). Conventional breeding for cassava improvement is constrained by its long life cycle, high level of heterozygosity, poor flowering, and seed set and inbreeding depression (Ceballos et al., 2004). CRISPR/Cas9-targeted genome editing therefore offers important new opportunities for addressing pre- and post-harvest constraints affecting cassava production and utilization. To evaluate the potential of CRISPR/Cas9 in a new species such as cassava, a reproducible system for design, construction, and delivery of Cas9/gRNA reagents must be developed and validated. We report here an efficient CRISPR/Cas9-mediated targeting of *Phytoene desaturase* (*PDS*) in two cassava cultivars. The results reported set the groundwork for future studies of gene function and trait development using the CRISPR/Cas9 gene-editing technology in this important staple food crop.

## Materials and Methods

### *In Silico* Identification of Cassava *Phytoene desaturase* and CRISPR/Cas9/gRNA Vector Construction

**FIGURE 1:**
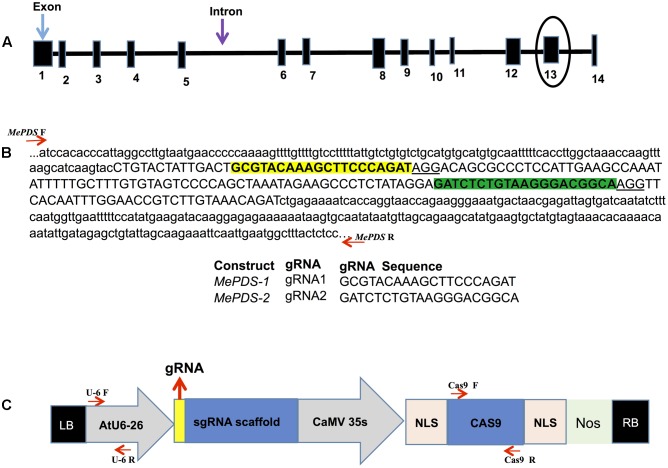
Schematic representations of the cassava *MePDS* target gene, location of the gRNAs, and CRISPR/Cas9 gene-editing construct. **(A)** Structural organization of the *MePDS* gene. Exons and introns are shown as boxes and lines, respectively, with the number of exons indicated below boxes. The gRNA where designed from the encircled exon 13. **(B)** Schematic representation of target region showing the sequences and location of the two 20 bp gRNAs. The sequence shown is a reverse complement of *MePDS* from cultivar AM560-2 (Phytozome 11, cassava4.1_004359m.g). gRNA1 and gRNA2 are highlighted in yellow and green, respectively, within exon 13 shown as upper case and surrounding intron sequences appears as lower case. Positions of forward (F) and reverse (R) primers flanking the target region in *MePDS* are indicted with red arrows, respectively. **(C)** Schematic of the CRISPR/Cas9 binary vector pCAMBIA2300_CR3-EF used for stable *Agrobacterium*-mediated transformation of cassava. *Arabidopsis thaliana* promoter (AtU6-26) drives expression of each gRNA used to target *MePDS*, with the gRNA ligated at the position indicated by the yellow box aided by the *Bsa1* restriction site. The Cauliflower mosaic virus promoter (CaMV 35S) drives expression of the Cas9 gene which together with inserted gRNA induce mutations in target region of *MePDS* gene; NLS, nuclear localization signal; Nos, Nos terminator; LB, left border; RB, right border. Position of forward (F) and reverse (R) primers used for amplifying respective regions of cassette are shown by red arrows.

BLAST was used to search the reference cassava genome in Phytozome 11 (version 4.1)^1^ for nucleotide sequences homologous to the *Arabidopsis thaliana PDS* gene (*AtPDS*: NM117498). A single copy gene, here referred to as *MePDS* (Manes.05G193700/cassava4.1_004359m.g), was identified. The CRISPR-P program (version 1)^2^ was employed to design gRNAs. Two target sequences were selected by initially identifying the NGG protospacer adjacent motif (PAM) sequence essential for *Streptococcus pyogenes* Cas9, followed by 20 nucleotides upstream of the PAM sequence for use as the spacer in the gRNA to target *MePDS.* Two gRNAs chosen were located in the 13^th^ exon, each having a G at their 5′ end to enhance gRNA transcription in plant cells using the U6-26 promoter when transcribed as single molecule with gRNA scaffold (**Figures 1A,C**).

A pair of complementary DNA oligonucleotides were synthesized (Integrated DNA Technologies^3^) and hybridized to form a duplex which has compatible ends with the *BsaI*-digested empty vector (pCAMBIA2300_CR3-EF, provided by B. Staskawicz, University of California, Berkeley, CA, United States). The empty vector construct contained Cas9 coding sequence from *S. pyogenes* and gRNA scaffold with a *BsaI* restriction site for insertion of a single gRNA. The included *npt*II marker gene aids in selection of transgenic cells during regeneration process. The 35S Cauliflower mosaic virus promoter was utilized to drive expression of Cas9, while the U6-26 promoter from Arabidopsis drove expression of the gRNAs (**Figure 1C**). The presence of the inserted gRNA and stability of the final constructs was confirmed by sequencing. The two binary vectors, *MePDS-1* and *MePDS-2*, each carrying one gRNA, were transformed into *Agrobacterium* strain LBA 4404 by electroporation.

### Cassava Transformation and Visual Confirmation of *PDS* Mutants

Friable embryogenic callus (FEC) was generated from cassava cultivars 60444 and TME 204 and used as target tissue for transformation with the gene-editing constructs *MePDS-1* and *MePDS-2*, plus empty vector (EV) and green fluorescent protein (GFP) controls (Taylor et al., 2012; Chauhan et al., 2015). Regenerated cotyledon-stage somatic embryos and whole plantlets with altered *PDS* activities were visually discriminated from control tissues under a dissection microscope. Plants were established in the greenhouse by transfer to Fafard 51 soilless potting medium (Conrad Fafard, Inc., Agawam, MA, United States) before moving them to the open bench and grown at a temperature of 26°C/25°C (day/night) with 60–90% relative humidity for 3 weeks to reach approximately 8 cm in height, at which time they were transferred to a glasshouse maintained at a temperature of 32°C/27°C (day/night) and 70–95% relative humidity (Taylor et al., 2012).

### Detection of CRISPR/Cas9 Induced Mutations

Leaf tissue was collected from rooted *in vitro* plantlets. Approximately 0.1 g of leaf tissue was placed in a 2-ml screw cap tube containing ceramic beads and crushed to fine powder using a Fast-Prep 24 (MP Biomedicals, Solon, OH, United States) set at 4.0 m/s for 30 s twice. Genomic DNA was extracted using cetyltrimethylammonium bromide (CTAB) protocol (Doyle and Doyle, 1990) and treated with RNase, DNase-free kit (Roche Diagnostics GmbH, Indianapolis, IN, United States) to remove RNA.

Putatively edited plant lines were subjected to polymerase chain reaction (PCR) to confirm integration of the T-DNA using 200 ng genomic DNA as a template and primers specific for the Cas9 gene, U6-26 promoter, and gRNA scaffold (**Table 1**), under the following conditions: 98°C for 5 min; 34 cycles of 98°C for 10 s, 65°C for 20 s, 72°C for 30 s followed by final extension of 72°C for 5 min. Genomic DNA template was used for PCR amplification of a 504-bp fragment spanning the targeted *MePDS* sequence. Briefly, 200 ng of DNA was added to Phusion DNA Polymerase Master Mix (New England Biolabs^4^) in a 25 μl reaction volume and PCR performed at 98°C for 5 min; 34 cycles of 98°C for 10 s, 64°C for 20 s, 72°C for 30 s, followed by final extension at 72°C for 5 min. A single primer pair was designed to amplify both target sites based on the *MePDS* gene sequence from reference genotype AM560-2 in Phytozome 11, version 4.1. PCR products were resolved on a 1.5% agarose gel, excised, and amplicons purified using the QIAquick gel extraction kit (Qiagen Group), before cloning into pCR Blunt-II TOPO vector (Life Technologies^5^). Between 5 and 10 single colonies per plant line were subjected to Sanger sequencing using M13F and M13R primers to characterize CRISPR/Cas9 induced mutations. Sequences were aligned with the wild-type reference sequence of *MePDS* gene using the MUSCLE alignment program of EMBL-EBI^6^. The mutation frequency in transgenic plants was calculated by expression of plant lines with phenotypic alterations or mutated sequences as a percentage of total plant lines examined.

**Table 1:** Primers used to confirm integration of T-DNA and integrity of *MePDS* target sequence.

Primer	Forward sequence	Reverse sequence	Product size (bp)
MePDS	GGAGAGTAAAGCCATTCAATTG	ATCCACACCCATTAGGCCTTG	504
Cas9 Gene	GCTGGGCCGTGATCACCGAC	CACTCTCAGGATGTCGCTCAGC	900
U6 Promoter	CAGGAAACAGCTATGACCATG	CATGTTGACCTGCAGGCA	700


## Results

The nucleotide sequence of *A. thaliana PDS* (*AtPDS*: NM117498) was used as bait to search for homologous sequences within the reference cassava genome in Phytozome 11 (version 4.1) and a single copy of *MePDS* identified. The genomic sequence of *MePDS* is 13,130 nucleotides in size, comprising 14 exons and 13 introns (**Figure 1A**), with 1,743 nucleotides of transcript sequence coding for 580 amino acids. *AtPDS* (NP_193157.1) and *MePDS* (Manes.05G193700.1) are 85% identical at the protein level, suggesting a conserved function in cassava. In order to disrupt *MePDS*, two distinct gRNAs were designed as predicted by the CRISPR-P program to have the highest efficacy and least off-target potential. The gRNAs were cloned into two separate binary vectors (*MePDS-1 and MePDS-2*) carrying the Cas9 gene (**Figure 1B**), Arabidopsis U6-26 promoter and gRNA scaffold (**Figure 1C**), and delivered by *Agrobacterium* into embryogenic cells of cassava.

### *PDS* Edited Plants Exhibit Dwarf and Albino Phenotypes

**FIGURE 2:**
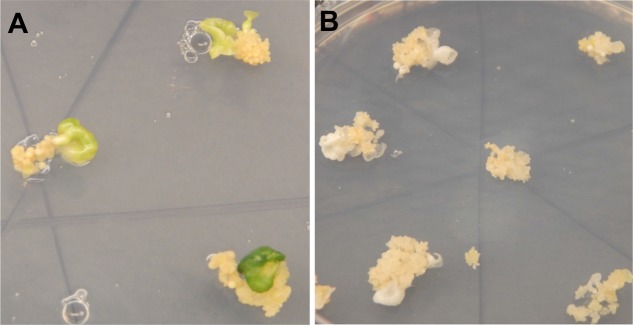
Regenerating cassava somatic embryos after CRISPR/Cas9-mediated mutation of *MePDS*. **(A)** Fully green cotyledon stage embryos regenerating from non-edited wild-type tissues of cv. 60444. **(B)** Regenerating albino cotyledons with CRISPR/Cas9 edited *MePDS.*

A total of 103 independent lines of regenerating somatic embryos were recovered from cv. 60444 and 85 from cv. TME 204 after selection on antibiotic-containing medium (Chauhan et al., 2015) (**Table 2**). Visual indication of altered *MePDS* function was first apparent as albino or partial albino cotyledon-stage somatic embryos regenerating on selection medium (**Figure 2**). This phenotype was observed in 100/103 (97.1%) and 84/85 (98.9%) somatic lines of cv. 60444 and TME 204 transgenic for *MePDS-1* and *MePDS-2*, respectively. Both constructs were equally effective for producing the mutated phenotype. A total of 47% of the somatic embryo lines from cv. 60444 and 22% from cv. TME 204 germinated to produce plantlets, generating 58 and 25 independent transgenic plant lines from 60444 and TME 204, respectively.

**Table 2:** Production and recovery of albino plants after CRISPR/Cas9-mediated gene editing of *MePDS*.

	60444					TME 204				
		
Construct	Somatic embryo lines	Plant lines recovered	Fully albino lines	Partially albino lines	Fully green lines	Somatic embryo lines	Plant lines recovered	Fully albino lines	Partially albino lines	Fully green lines
*MePDS-1*	45	23	2	19	2	38	9	7	2	0
*MePDS-2*	58	27	10	16	1	47	10	6	3	1
EV control	11	5	0	0	5	20	4	0	0	4
WT	8	3	0	0	3	9	2	0	0	2
**Total**	**122**	**58**	**12**	**35**	**11**	**114**	**25**	**13**	**5**	**7**


*EV, empty vector control; WT, non-transgenic wild-type control.*

A diversity of albino phenotypes was observed in the regenerated plants, ranging from full albinos with total loss of green coloration, to partial albinos displaying a mixture of green and white tissues on the same plantlet (**Figures 2**, **4**). In all cases, non-edited wild-type, EV and GFP-expressing control plants remained fully green. Both gRNAs generated albino mutants with high frequencies ranging from 90% for *MePDS-2* to 100% for *MePDS-1* in cv. TME 204 (**Figure 6A**). Mutant plants characterized by full or partial albino phenotypes displayed diminished growth rates, shorter petioles, and reduced leaf area compared to the non-edited control equivalents (**Figures 4**, **5**). Albino plant lines with greatly or totally diminished green tissue (**Figures 4**, **5A**) did not survive more than two to three successive *in vitro* propagation cycles. A selected set of partial albino and control plants was transferred to soil, where the dwarf phenotype and albino nature were maintained when grown under greenhouse conditions. In contrast, control EV and GFP-expressing control plants were fully green and showed robust growth (**Figure 5**).

### Molecular Analysis of CRISPR/Cas9 Induced Mutations in *MePDS* Gene

**FIGURE 3:**
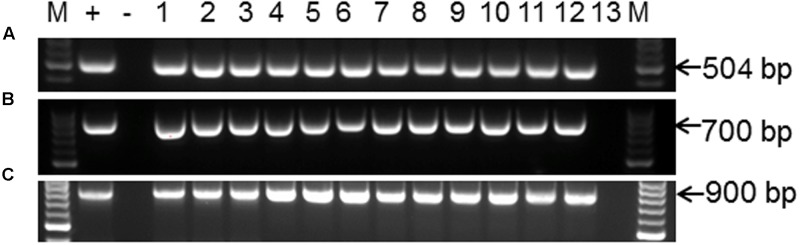
PCR amplification and detection of CRISPR/Cas9 T-DNA integration and the *MePDS* target sequence. **(A)** PCR amplification for 504 bp of the *MePDS* target region **(B)** confirmation of CRISPR/Cas9 presence by detection of the Cas9 gene. **(C)** The U6-26 promoter and gRNA scaffold. The expected band sizes are shown on the left as 504, 900, and 700 bp for **(A–C)**, respectively. M is molecular size marker. For **(A)**, + is an edited positive reference sample from 60444 confirmed by sequencing, lanes 1–11 display PCR amplicons from *MePDS* edited plant lines lanes, lane 12 is a wild-type plant, and lane 13 is water control. For **(B,C)** + is plasmid DNA containing the binary vector pCAMBIA2300, – is a no template control, lanes 1–12 are PCR products from selected *MePDS* edited plant lines, and lane 13 a wild-type control.

Polymerase chain reaction (PCR) was performed on genomic DNA extracted from regenerated plants to detect the presence of the T-DNA and to amplify the targeted *MePDS* gene (**Table 1**). As expected, all albino and EV plant lines tested positive for presence of the Cas9 gene, U6-26 promoter, and gRNA scaffold (**Figures 3B,C**). A 504 bp fragment corresponding to the target region of the *MePDS* gene in cassava was amplified (**Figure 3A**), and amplicons from 38 plant lines (19 each for 60444 and TME 204) were cloned and 241 clones sequenced (**Table 3**). All full or partial albino plants were found to carry mutations in their *MePDS* gene, with the *MePDS-1* and *MePDS-2* constructs equally efficacious for inducing mutations in the two cultivars tested (**Table 3**). Both INDELs and substitutions were observed. Regardless of the construct and cultivar tested, substitutions occurred more frequently than insertions and deletions (**Figure 6B**). Constructs *MePDS-1* and *MePDS-2* directed insertion or substitution of different nucleotides. In cv. 60444, both constructs induced insertion and substitution of nucleotide ‘A’ compared to substitution of nucleotides ‘CA’ and ‘T’ in cv. TME 204. The longest deletion detected in cv. 60444 was 16 bp, compared to 101 bp observed in cv. TME 204 plant line 4 (Supplementary Figure S1). Examination of the 504 bp *MePDS* target sequence after Sanger sequencing revealed 1 bp nucleotide substitutions and INDELs upstream of the 5′ and or downstream of the 3′ *MePDS* target region for both constructs *MePDS-1* and *MePDS-2*. We observed a single nucleotide polymorphism (SNP), which overlapped with *MePDS-2* gRNA (‘C’ substituted for ‘G’) targeting the *MePDS* region of cv. TME 204, but this did not negatively affect CRISPR/Cas9 editing of *MePDS.*

**Table 3:** Categories of mutations detected after CRISPR/Cas9-mediated targeting of cassava *MePDS* gene.

	60444					TME 204				
		
Construct	Plant lines analyzed	Clones analyzed	Homozygous mono-allelic	Homozygous bi-allelic	Heterozygous	Plant lines analyzed	Clones analyzed	Homozygous mono-allelic	Homozygous bi-allelic	Heterozygous
*MePDS-1*	9	54	1	1	7	9	71	0	3	6
*MePDS-2*	10	55	0	0	10	10	61	0	1	9
**Total**	**19**	**58**	**1**	1	**17**	**19**	**132**	**0**	**4**	**15**


The approach developed by Fan et al. (2015) in populus was used to classify plant lines as putatively homozygous or heterozygous for the induced mutations with slight modification. Sequences derived from albino mutants were aligned and compared with the reference wild-type sequence of *MePDS*. Mutant lines in which all clones examined carried one identical type of mutation were classified as putatively homozygous mono-allelic, while mutants whose clones displayed two identical types or patterns of mutations were considered to be putatively homozygous bi-allelic. Mutants categorized as heterozygous were chimeric, having at least three types or patterns of mutations and/or wild-type sequence present in the same plant. Based on this scheme, 1/19 plant lines (5.23%, line 9) of cv. 60444 was putatively homozygous mono-allelic since all five clones carried two substitutions (**Figure 7**). Further scrutiny revealed that 1/19 plant lines (5.23%, line 8) of cv. 60444 and 4/19 plant lines (21.1%, lines 2, 6, 7, and 9) of cv. TME 204 were putatively homozygous bi-allelic (**Table 3**); 17/19 (89.5%) of cv. 60444 and 15/19 (78.9%) of cv. TME 204 mutant plant lines were categorized as heterozygous (**Table 3**). Assessment of sequence and phenotypic data further indicated that the majority of full albino plant lines edited with *MePDS-1* were putatively homozygous for mutations at *MePDS* (**Table 3**). Plant line 5 of cv. TME 204 which remained fully green showed sequence alteration in only one of the two *MePDS* alleles (**Figure 7**). The altered sequence (putatively homozygous or heterozygous) and albino (full or partial albino) frequency data agree with over 90% of CRISPR/Cas9 directed mutation frequency (**Figure 6A**). When the 504 bp *MePDS* target sequence was examined for the presence of off targets, Sanger sequencing revealed 1 bp nucleotide substitutions and INDELs upstream of the 5′ and or downstream of the 3′ target region. In most cases, such off-target effects occurred next to a random PAM site NGG or NCC within the *MePDS* gene.

## Discussion

CRISPR/Cas9 genome-editing technology has become an effective tool for basic research and trait development in crop plants (Pennisi, 2013; Zhang et al., 2017). To date, however, no such capacity has been reported for the tropical crop cassava. Efficient gene editing in this important staple food crop would present new opportunities to address biotic and abiotic constraints in cassava production and post-harvest utilization (Bart and Taylor, 2017). Toward this end, CRISPR/Cas9 was employed to target the *PDS* gene in cassava. *PDS* codes for key enzymes in the carotenoid biosynthesis pathway (Qin et al., 2007). Defects in *PDS* gene function cause dwarfism and albino phenotypes in Arabidopsis due to impaired chlorophyll, carotenoid, and gibberellin biosynthesis (Qin et al., 2007). Reports of CRISPR/Cas9 targeting of *PDS* in rice, apple, sweet orange, and populus resulted in similar phenotypic aberrations (Jia and Wang, 2014; Zhang et al., 2014; Fan et al., 2015; Nishitani et al., 2016). Deliberate alteration of the *PDS* gene was therefore chosen as a visual marker to reveal efficacious application of CRISPR/Cas9-targeted genome editing in cassava. *PDS* offers an attractive target for developing gene-editing techniques due to its monogenic nature and the easily assessed albino phenotype resulting from its mutation (Jia and Wang, 2014; Wang et al., 2015; Nishitani et al., 2016).

**FIGURE 4:**
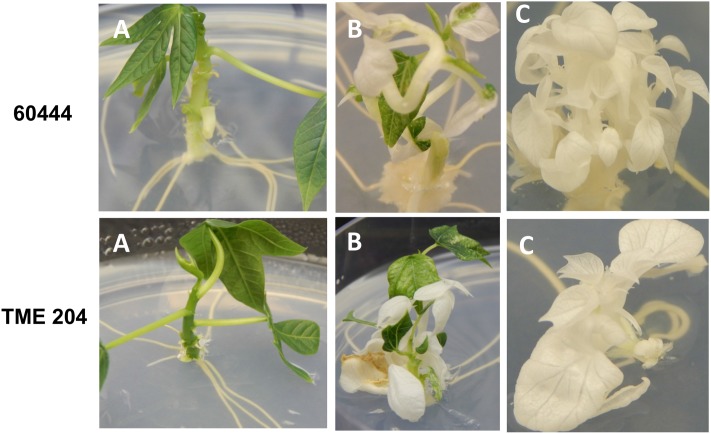
Phenotypic diversity of CRISPR/Cas9 induced *MePDS* mutations in *in vitro* cassava plantlets. Upper panel shows cv. 60444, and lower panel, cv. TME 204. **(A)** Non-edited wild-type control plants with fully green shoots, **(B)** chimeric albino plant showing a mixture of green and white shoot tissues, and **(C)** fully albino plants.

**FIGURE 5:**
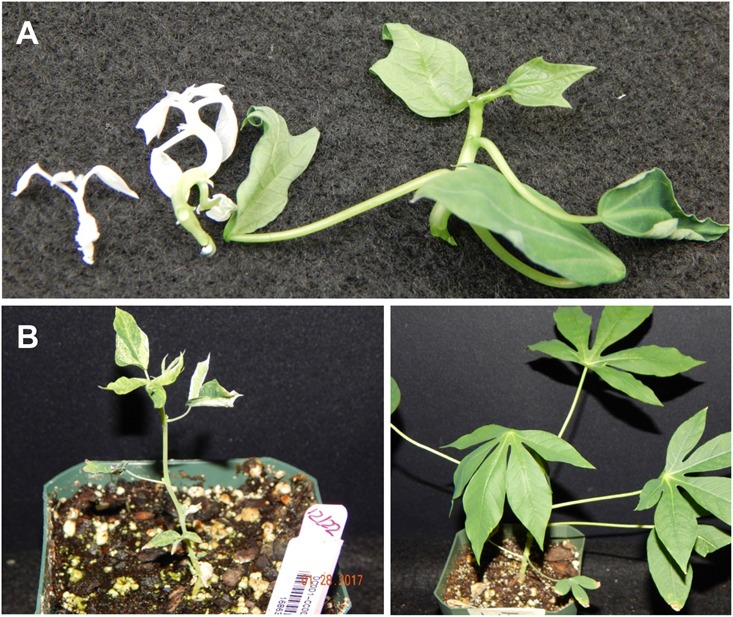
Effects of CRISPR/Cas9 directed alteration of *MePDS* gene function on phenotypes of regenerated cassava plants. **(A)** Dwarf *in vitro* plants of cv. TME 204 edited with *MePDS-2* with green non-modified plant on the right, **(B)** greenhouse grown plants of cv. 60444 edited with *MePDS-1* characterized by stunted growth, shorter petioles, and reduced leaf area compared to the robust fully green non-edited wild-type control on the right.

In order to maximize our chance of success, two gRNAs were designed to interrupt *MePDS* function by targeting exon 13 in cassava cultivars 60444 and TME 204. The 13^th^ exon was chosen because CRISPR-P output indicated that it provided optimal gRNAs starting with ‘G’ for use with the U6 promoter for achieving successful gene editing. In addition, previous studies report that mutations to the *PDS* gene at any location translate into phenotypic alteration. Existing genetic transformation systems (Taylor et al., 2012; Chauhan et al., 2015) were employed to integrate the CRISPR/Cas9 tools into embryogenic cells. Albino phenotypes were recovered from both cultivars after transformation with two gRNA constructs (**Figures 2**, **4**, **5** and **Table 2**). Albino somatic embryos were visualized as early as 8 weeks after transformation and could be regenerated to plantlets displaying full and partial albino characteristics (**Figures 2**, **4**, **5**), in a manner similar to that reported in other plant species (Qin et al., 2007; Jia and Wang, 2014; Fan et al., 2015; Nishitani et al., 2016). Recovery of the albino mutants occurred at unexpectedly high frequencies of 93–95% of the regenerated plant lines of cvs. 60444 and TME 204, respectively. The albino phenotypes observed in the current study resulted from loss of *MePDS* function generated by action of the CRISPR/Cas9 reagents. The observed diversity and degree of the albino phenotype recorded here could have resulted from differential timing of the targeted mutation. Due to the integrated nature of the editing tools, editing will continue from CRISPR/Cas9 expressing cassettes within a given cell until the target site is mutated. This can result in chimeric plants consisting of cells with non-modified genomes, and populations of cells carrying different gene edited events. The exact pattern of these would depend on timing of CRISPR/Cas9-mediated editing in relation to the plant regeneration process. In rice, for example, the occurrence of full albino mutants totally lacking chlorophyll and partial albinos characterized by a mixture of albino and green tissues was attributed to induction of mutations before and after first cell division (Zhang et al., 2014).

**FIGURE 6:**
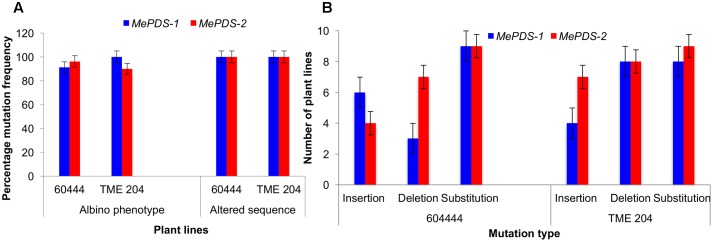
CRISPR/Cas9 induced mutation frequency and types. **(A)** Percentage frequency of mutation based on occurrence of albino phenotypes and recovered altered sequence in plant lines of cv. 60444 and cv. TME 204. Albino mutation frequency was calculated as number of germinated albino plants lines expressed as percentage of total number of plant lines generated. Frequency of plants with altered sequence was obtained by expressing number of plant lines with sequence alteration as a ratio of total plant lines sequenced. **(B)** Number of plant lines with the various induced mutations following alignment of wild-type sequence with the test sequences. *MePDS-1* and *MePDS-2* are plasmids carrying the gRNAs used to target *MePDS* gene.

**FIGURE 7:**
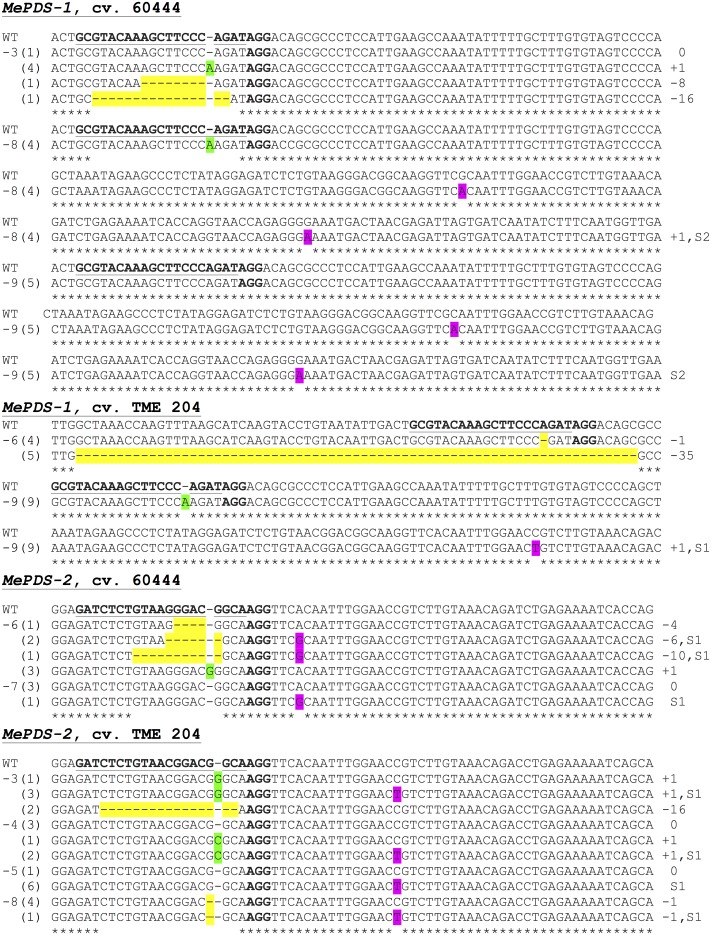
Sequence-based detection of mutations induced by CRISPR/Cas9 constructs *MePDS-1* and *MePDS-2* in cultivars 60444 and TME 204. For each plant line characterized, PCR was used to amplify across the target region. The PCR product was cloned into pCR Blunt-II TOPO vector and transformed into *E. coli*. Multiple individual colonies were analyzed via Sanger sequencing to detect mutations near the target site. Aligned sequence data is shown for 11 representative mutant plant lines. The target region of *MePDS* is underlined and bolded in the wild-type (WT) reference sequence, with the protospacer adjacent motif (PAM) in bold but not underlined. Sequences from each plant line (-3, -8, etc.,) are shown below the WT sequence. The number of colonies with a specific sequence pattern is indicated in parentheses. Deletions are highlighted in yellow, insertions are highlighted green, and substitutions are highlighted in pink. Mutation type insertion (+), deletion (–), or substitution (S) and size are indicated at the right side of the panel.

Molecular analysis was performed on albino and green plant lines, with multiple clones sequenced per plant line to identify mutations within one or both *MePDS* alleles. Sequence data indicated the presence of INDELs and substitutions in all (100%) of the 38 (19 from each cultivar) regenerated plant lines examined at the DNA level. Irrespective of cultivar and construct used, INDELs mostly occurred 1–3 bp upstream from the PAM site. Predominantly single nucleotide insertions and substitutions (**Figure 7**) were detected in *MePDS* resulting from both gRNA constructs. However, larger deletions, including 1 of 101 bp in TME 204, were also observed (Supplementary Figure S1). The INDELs recorded here could have resulted from NHEJ repair following integration of our constructs *MePDS-1* and *MePDS-2* as previously reported in apple and populus following CRISPR/Cas9-targeted mutation of the PDS gene (Fan et al., 2015; Nishitani et al., 2016). Observation of single nucleotide substitutions upstream or downstream of the target region (**Figures 6B**, **7** and Supplementary Figure S1) implies activation of HR pathway to repair DSBs created in *MePDS*. Simultaneous triggering of HR and NHEJ repair pathways proceeding integration of CRISPR/Cas9 reagents was documented earlier in apple, *N. benthamiana*, and cotton (Li et al., 2013; Nishitani et al., 2016; Gao et al., 2017). We also recovered wild-type sequences from some colonies derived from plants that clearly showed altered phenotype (**Figure 7**). This finding is similar to data reported for CRISPR/Cas9 gene targeting in *N. benthamiana* and rice (Nekrasov et al., 2013; Zhang et al., 2014). Gene edited TME 204 plant line #4, transgenic for *MePDS-1*, returned three clones with double nucleotide substitutions (CA) (Supplementary Figure S1). This rare occurrence seems to indicate that CRISPR/Cas9 can direct differing frequency and types of mutations. Induction of high mutation rates by *MePDS-2* gRNA that carried a single bp mismatch with the cv. TME 204 target sequence was common. This finding is not surprising in light of a similar recent report in which a target sequence with a single nucleotide difference was deliberately chosen to successfully induce CRISPR/Cas9 directed mutation in homologous *Chloroplastos alterados* 1 genes of cotton (GhCLA1) (Gao et al., 2017). Four transgenic plant lines out of the 69 (4/69, 5.8%) examined displayed unexpected visible green phenotype like WT and EV controls. However, sequence data showed presence of nucleotide substitutions in one allele of *MePDS* in these plants (**Figure 7**). This phenomenon is consistent with results obtained in citrus, Arabidopsis, and *N. benthamiana* (Li et al., 2013; Nekrasov et al., 2013; Shan et al., 2013; Jia and Wang, 2014), and could result from presence of very low populations of photo-bleached *PDS* cells among non-modified tissues (Li et al., 2013). Alternatively, the presence of a single, fully functional allele may be sufficient to ensure functionality of *MePDS*. Possibly because of the high mutation efficiency achieved, we simultaneously induced putatively homozygous (up to 21.1% in TME 204) and heterozygous (up to 89.5% in 60444) mutations in *MePDS* gene in these T_0_ events. All putatively homozygous, mono-allelic plants showed full albino phenotypes while putatively homozygous, bi-allelic plants were either full or partial albinos, with putatively heterozygous counterparts displaying partial albino phenotypes.

The very high efficiency of mono and bi-allelic gene editing achieved from two constructs across both cultivars studied equates with similar reports in potato (83%) (Wang et al., 2015) and cotton (98–100%) (Gao et al., 2017; Li et al., 2017). The surviving edited mutants showed persistence of altered phenotype across micropropagation cycles and under greenhouse conditions. These results indicate significant potential for successful application of gene-editing technologies for the development of cassava with improved farmer and consumer traits. There is need, however, to further develop CRISPR/Cas9 gene-editing capacity for this crop. It is considered that the high efficiencies achieved here result, at least in part, from integration of the CRISPR/Cas9 and gRNA cassette. Continuous expression of the editing tools maximizes opportunity for production of mutations at the target site. Although a powerful tool for basic research, such an approach is not optimal for developing enhanced germplasm with value for cassava farmers. Segregating away the integrated T-DNA by sexual crossing in cassava is possible, but is not desirable due to the crop’s heterozygous nature and associated in-breeding depression. It is important, therefore, to develop genome-editing capacity for cassava that does not require integration of the CRISPR tools. Such techniques include direct delivery into totipotent cells and use of ribonucleoprotein (RNP) (Woo et al., 2015; Zhang et al., 2017) as alternatives to *Agrobacterium*-mediated transgenesis. The expanding range of alternatives to Cas, such as Cpf1 (Zetsche et al., 2015), also brings increased flexibility for targeting genes, transcription factors, and their control elements. The *MePDS* knockout system described here generates easily detectable mutated events in a relatively short time frame of 6–8 weeks, and due to direct visualization of the phenotype, does not require sequencing to confirm editing at the target. It therefore provides a valuable method to facilitate rapid assessment and optimization of CRISPR/Cas9 and other genome-editing technologies in cassava.

## Author Contributions

JO designed the experiments, produced, and analyzed the gene edited plants and wrote the manuscript. TA and II interpreted the data and edited the manuscript. RB designed the analysis and interpreted sequencing data, wrote and edited the manuscript. DN designed the experiments and edited the manuscript. NT supervised the design of experiments and analysis of edited plants, wrote and edited the manuscript.

## Conflict of Interest Statement

The authors declare that the research was conducted in the absence of any commercial or financial relationships that could be construed as a potential conflict of interest.

## Footnotes

1 https://phytozome.jgi.doe.gov/pz/portal.html

2 http://cbi.hzau.edu.cn/cgi-bin/CRISPR

3 https://www.idtdna.com/site

4 https://www.neb.com/

5 https://www.thermofisher.com/us/en/home.html

6 http://www.ebi.ac.uk/Tools/msa/muscle/

## References

[B1] Bart R. S. Taylor N. J. (2017). New opportunities and challenges to engineer disease resistance in cassava, a staple food of African small-holder farmers. *PLOS Pathog.* 13:e1006287. 10.1371/journal.ppat.1006287 28493983 PMC5426740

[B2] Brooks C. Nekrasov V. Lippman Z. B. Van Eck J. (2014). Efficient gene editing in tomato in the first generation using the clustered regularly interspaced short palindromic repeats/CRISPR-associated9 system. *Plant Physiol.* 166 1292–1297. 10.1104/pp.114.247577 25225186 PMC4226363

[B3] Ceballos H. Iglesias C. A. Perez J. C. Dixon A. G. (2004). Cassava breeding: opportunities and challenges. *Plant Mol. Biol.* 56 503–516. 15630615 10.1007/s11103-004-5010-5

[B4] Ceballos H. Kawuki R. S. Gracen V. E. Yencho G. C. Hershey C. H. (2015). Conventional breeding, marker-assisted selection, genomic selection and inbreeding in clonally propagated crops: a case study for cassava. *Theor. Appl. Genet.* 128 1647–1667. 10.1007/s00122-015-2555-4 26093610 PMC4540783

[B5] Chandrasekaran J. Brumin M. Wolf D. Leibman D. Klap C. Pearlsman M. (2016). Development of broad virus resistance in non-transgenic cucumber using CRISPR/Cas9 technology. *Mol. Plant Pathol.* 17 1140–1153. 10.1111/mpp.12375 26808139 PMC6638350

[B6] Chauhan R. D. Beyene G. Kalyaeva M. Fauquet C. M. Taylor N. (2015). Improvements in *Agrobacterium*-mediated transformation of cassava (*Manihot esculenta* Crantz) for large-scale production of transgenic plants. *Plant Cell Tiss. Organ Cult.* 121 591–603. 10.1007/s11240-015-0729-z

[B7] Curtin S. J. Voytas D. F. Stupar R. M. (2012). Genome engineering of crops with designer nucleases. *Plant Genome* 5 42–50. 10.3835/plantgenome2012.06.0008 27645899

[B8] Ding Y. Li H. Chen L. L. Xie K. (2016). Recent advances in genome editing using CRISPR/Cas9. *Front. Plant Sci.* 7:703. 10.3389/fpls.2016.00703 27252719 PMC4877526

[B9] Doudna J. A. Charpentier E. (2014). Genome editing. The new frontier of genome engineering with CRISPR-Cas9. *Science* 346:1258096. 10.1126/science.1258096 25430774

[B10] Doyle J. J. Doyle J. L. (1990). Isolation of plant DNA from fresh tissue. *Focus* 12 13–15.

[B11] Fan D. Liu T. Li C. Jiao B. Li S. Hou Y. (2015). Efficient CRISPR/Cas9-mediated targeted mutagenesis in populus in the first generation. *Sci. Rep.* 5:12217. 10.1038/srep12217 26193631 PMC4507398

[B12] Gao W. Long L. Tian X. Xu F. Liu J. Singh P. K. (2017). Genome editing in cotton with the CRISPR/Cas9 system. *Front. Plant Sci.* 8:1364. 10.3389/fpls.2017.01364 28824692 PMC5541054

[B13] Jia H. Wang N. (2014). Targeted genome editing of sweet orange using Cas9/sgRNA. *PLOS ONE* 9:e93806. 10.1371/journal.pone.0093806 24710347 PMC3977896

[B14] Jiang W. Zhou H. Bi H. Fromm M. Yang B. Weeks D. P. (2013). Demonstration of CRISPR/Cas9/sgRNA-mediated targeted gene modification in Arabidopsis, tobacco, sorghum and rice. *Nucleic Acids Res.* 41 e188. 10.1093/nar/gkt780 23999092 PMC3814374

[B15] Johnson R. D. Jasin M. (2001). Double-strand-break-induced homologous recombination in mammalian cells. *Biochem. Soc. Trans.* 29 196–201. 11356153 10.1042/0300-5127:0290196

[B16] Joung J. K. Sander J. D. (2013). TALENs: a widely applicable technology for targeted genome editing. *Nat. Rev. Mol. Cell Biol.* 14 49–55. 10.1038/nrm3486 23169466 PMC3547402

[B17] Li C. Unver T. Zhang B. (2017). A high-efficiency CRISPR/Cas9 system for targeted mutagenesis in cotton (*Gossypium hirsutum* L.). *Sci. Rep.* 7:43902. 10.1038/srep43902 28256588 PMC5335549

[B18] Li J. F. Norville J. E. Aach J. Mccormack M. Zhang D. Bush J. (2013). Multiplex and homologous recombination-mediated genome editing in *Arabidopsis* and *Nicotiana benthamiana* using guide RNA and Cas9. *Nat. Biotechnol.* 31 688–691. 10.1038/nbt.2654 23929339 PMC4078740

[B19] Nekrasov V. Staskawicz B. Weigel D. Jones J. D. Kamoun S. (2013). Targeted mutagenesis in the model plant *Nicotiana benthamiana* using Cas9 RNA-guided endonuclease. *Nat. Biotechnol.* 31 691–693. 10.1038/nbt.2655 23929340

[B20] Nishitani C. Hirai N. Komori S. Wada M. Okada K. Osakabe K. (2016). Efficient genome editing in apple using a CRISPR/Cas9 system. *Sci. Rep.* 6:31481. 10.1038/srep31481 27530958 PMC4987624

[B21] Pennisi E. (2013). The CRISPR craze. *Science* 341 833–836. 10.1126/science.341.6148.833 23970676

[B22] Puchta H. Dujon B. Hohn B. (1996). Two different but related mechanisms are used in plants for the repair of genomic double-strand breaks by homologous recombination. *Proc. Natl. Acad. Sci. U.S.A.* 93 5055–5060. 8643528 10.1073/pnas.93.10.5055 PMC39405

[B23] Qin G. Gu H. Ma L. Peng Y. Deng X. W. Chen Z. (2007). Disruption of phytoene desaturase gene results in albino and dwarf phenotypes in *Arabidopsis* by impairing chlorophyll, carotenoid, and gibberellin biosynthesis. *Cell Res.* 17 471–482. 10.1038/cr.2007.40 17486124

[B24] Shan Q. Wang Y. Li J. Zhang Y. Chen K. Liang Z. (2013). Targeted genome modification of crop plants using a CRISPR-Cas system. *Nat. Biotechnol.* 31 686–688. 10.1038/nbt.2650 23929338

[B25] Sonoda E. Hochegger H. Saberi A. Taniguchi Y. Takeda S. (2006). Differential usage of non-homologous end-joining and homologous recombination in double strand break repair. *DNA Repair* 5 1021–1029. 10.1016/j.dnarep.2006.05.022 16807135

[B26] Sun X. Hu Z. Chen R. Jiang Q. Song G. Zhang H. (2015). Targeted mutagenesis in soybean using the CRISPR-Cas9 system. *Sci. Rep.* 5:10342. 10.1038/srep10342 26022141 PMC4448504

[B27] Svitashev S. Schwartz C. Lenderts B. Young J. K. Mark Cigan A. (2016). Genome editing in maize directed by CRISPR-Cas9 ribonucleoprotein complexes. *Nat. Commun.* 7:13274. 10.1038/ncomms13274 27848933 PMC5116081

[B28] Symington L. S. Gautier J. (2011). Double-strand break end resection and repair pathway choice. *Annu. Rev. Genet.* 45 247–271. 10.1146/annurev-genet-110410-132435 21910633

[B29] Taylor N. Gaitan-Solis E. Moll T. Trauterman B. Jones T. Pranjal A. (2012). A High-throughput platform for the production and analysis of transgenic cassava (*Manihot esculenta*) plants. *Trop. Plant Biol.* 5 127–139. 10.1007/s12042-012-9099-4

[B30] Wang S. Zhang S. Wang W. Xiong X. Meng F. Cui X. (2015). Efficient targeted mutagenesis in potato by the CRISPR/Cas9 system. *Plant Cell Rep.* 34 1473–1476. 10.1007/s00299-015-1816-7 26082432

[B31] Woo J. W. Kim J. Kwon S. I. Corvalan C. Cho S. W. Kim H. (2015). DNA-free genome editing in plants with preassembled CRISPR-Cas9 ribonucleoproteins. *Nat. Biotechnol.* 33 1162–1164. 10.1038/nbt.3389 26479191

[B32] Zetsche B. Gootenberg J. S. Abudayyeh O. O. Slaymaker I. M. Makarova K. S. Essletzbichler P. (2015). Cpf1 is a single RNA-guided endonuclease of a class 2 CRISPR-Cas system. *Cell* 163 759–771. 10.1016/j.cell.2015.09.038 26422227 PMC4638220

[B33] Zhang H. Zhang J. Wei P. Zhang B. Gou F. Feng Z. (2014). The CRISPR/Cas9 system produces specific and homozygous targeted gene editing in rice in one generation. *Plant Biotechnol. J.* 12 797–807. 10.1111/pbi.12200 24854982

[B34] Zhang K. Raboanatahiry N. Zhu B. Li M. (2017). Progress in genome editing technology and its application in plants. *Front. Plant Sci.* 8:177. 10.3389/fpls.2017.00177 28261237 PMC5306361

