# Supplementary material for: Efficient CRISPR/Cas9 Genome Editing of Phytoene desaturase in Cassava
Source: Front Plant Sci. 2017 Oct 18;8:1780. doi: 10.3389/fpls.2017.01780 (PMC5651273; doi:10.3389/fpls.2017.01780)
Supplement: Supplementary file 1 [file Image_1.pdf]

Supplementary Material

Efficient CRISPR/CAS9 Genome Editing of *Phytoene desaturase* in Cassava

John Odipio, Titus Alicai, Ivan Ingelbrecht, Dmitri A. Nusinow, Rebecca Bart and Nigel J. Taylor*

***Correspondence:** Nigel Taylor: ntaylor@danforthcenter.org

Supplementary Figure S1. Sequenced-based detection of mutations induced by CRISPR/Cas9 construct *MePDS-1* in cultivar TME 204. PCR was used to amplify across the target region. The PCR product was cloned into pCR Blunt-II TOPO vector and transformed into *E. coli*. Multiple individual colonies were analysed via Sanger sequencing to detect mutations near the target site. Sequence alignment for six colonies was derived from mutant plant line 4 (-4). The target region of *MePDS* is underlined and bolded in the wild type reference sequence (WT), with the protospacer adjacent motif (PAM) in bold but not underlined. The number of colonies with a specific sequence pattern is indicated in parentheses. Deletions are highlighted in yellow and substitutions are highlighted in pink. Mutation type, deletion (-) or substitution (S) and size are indicated at the right side of the panel.

***MePDS-1*, cv. TME 204**

```
WT     GTAATATTGACTGCGTACAAAGCTTCCCAGATAGGACAGCGCCCTCCATTGAAGCCAAATATTTTTGCTTTGTGTAGTCCCCAGCTAAAT
-4(1)  GTAATATTGACTGCGTACAAAGCTTCCCAGATAGGACAGCGCCCTCCATTGAAGCCAAATATTTTTGCTTTGTGTAGTCCCCAGCTAAAT
  (1)  GTAATATTGACTGCGTACAAAGCTTCCCAGATAGGACAGCGCCCTCCATTGAAGCCAAATATTTTTGCTTTGTGTAGTCCCCAGCTAAAT
  (2)  GTACAATTGACTGCGTACAAAGCTTCCCAGATAGGACAGCGCCCTCCATTGAAGCCAAATATTTTTGCTTTGTGTAGTCCCCAGCTAAAT
  (1)  GTAATATTGACTGCGTACAAAGCTTCCCAGATAGGACAGCGCCCTCCA------------------------------------------
  (1)  GTACAATTGACTGCGTACAAAGCTTCCCAGATAGGACAGCGCCCTCCA------------------------------------------
       ***  *******************************************

WT     AGAAGCCCTCTATAGGAGATCTCTGTAACGGACGGCAAGGTTCACAATTTGGAACCGTCTTGTAAACAGACCTGAGAAAAATCAGCAGGT
-4(1)  AGAAGCCCTCTATAGGAGATCTCTGTAACGGAC-GCAAGGTTCACAATTTGGAACTGTCTTGTAAACAGACCTGAGAAAAATCAGCAGGT -1
  (1)  AGAAGCCCTCTATAGGAGATCTCTGTAACGGAC---AAGGTTCACAATTTGGAACCGTCTTGTAAACAGACCTGAGAAAAATCAGCAGGT -3
  (2)  AGAAGCCCTCTATAGGAGATCTCTGTAACGGAC---AAGGTTCACAATTTGGAACCGTCTTGTAAACAGACCTGAGAAAAATCAGCAGGT -3,S2
  (1)  -----------------------------------------------------------TTGTAAACAGACCTGAGAAAAATCAGCAGGT -101
  (1)  -----------------------------------------------------------TTGTAAACAGACCTGAGAAAAATCAGCAGGT -101,S2
                                                                  *******************************
```
